# In-depth analysis of cystic fibrosis cases caused by *CFTR* gene variation and research on the prediction and simulation of the impact on protein function

**DOI:** 10.3389/fped.2025.1574919

**Published:** 2025-05-13

**Authors:** Yuxia Shan, Ziwei Zhu, Xiaomei Liu, Lei Chi, Jianqin Zhang, Li Cheng, Tianyi Liu

**Affiliations:** ^1^ Department of Pediatric Respiratory, Dalian Women and Children’s Medical Group, Dalian, Liaoning, China; ^2^ Department of Pharmacy, Dalian Women and Children’s Medical Group, Dalian, Liaoning, China

**Keywords:** *CFTR*, molecular dynamics simulation, cystic fibrosis, gene mutation, gene detection, clinical diagnosis

## Abstract

**Background:**

Cystic fibrosis (CF) is caused by *CFTR* gene mutations. Its diagnosis mainly depends on genetic and sweat chloride tests, but the complexity of these mutations challenges diagnosis.

**Methods:**

This paper reports a new case of a Chinese child with cough and wheezing, suspected of having CF. Trio whole-exome sequencing for the pedigree was carried out to detect *CFTR* gene mutations. Five tools, namely Mutation Taster, PolyPhen-2, SIFT, FATHMM, and PROVEAN, were used to predict the impacts of mutations on protein function. AlphaFold was employed to predict protein structures, and GROMACS software was used to conduct stability analysis through molecular dynamics simulations.

**Results:**

The child was diagnosed with severe pneumonia, plastic bronchitis, and acute asthmatic bronchitis, with a high suspicion of CF. Whole-exome sequencing revealed compound missense mutations in the *CFTR* gene: c.1408 (exon 11) G>A (p.V470M) and c.650 (exon 6) A>G (p.E217G), both of which were homozygous mutations. Parental genetic tests showed that the father was heterozygous for the mutations, and the mother was heterozygous at the c.650 (exon 6) A>G locus and homozygous at the c.1408 (exon 11) G>A locus. The results obtained by different prediction tools varied, and molecular dynamics simulations indicated that these mutations significantly affected the stability of the CFTR protein.

**Conclusion:**

Analysis of this new case using multiple tools and computational chemistry simulations helps to further understand the impacts of mutations on CFTR protein function and the disease, offering novel insights into the diagnosis, treatment, and genetic counseling of CF caused by the complex and diverse mutations of the *CFTR* gene.

## 1 Introduction

Cystic fibrosis (CF) is a hereditary disorder caused by mutations in the Cystic Fibrosis Transmembrane Conductance Regulator (*CFTR*) gene (1). It manifests with a wide variety of clinical symptoms in the respiratory, digestive, and other systems, leading to a decrease in the quality of life of patients and potentially triggering life-threatening complications (2). Currently, approximately 35% of cystic fibrosis patients worldwide remain undiagnosed. Moreover, due to the complexity of CFTR gene mutations, the diagnostic process is fraught with challenges. Therefore, there is an urgent need for novel non-invasive auxiliary diagnostic methods (3).

This paper specifically reports a newly emerged case of compound missense mutations in the *CFTR* gene in China. The child presented to the hospital with cough and wheezing. After diagnosis, the child was found to have severe pneumonia, plastic bronchitis, and acute asthmatic bronchitis. The disease progressed rapidly, and there were recurrent infections. Genetic testing revealed that the child's *CFTR* gene harbored compound missense mutations: the base at position 650 in exon 6 was mutated from A to G (p.E217G), and the base at position 1408 in exon 11 was mutated from G to A (p.V470M). Notably, the child's father had a normal phenotype and was heterozygous for the mutations at both the p.V470M locus and the p.E217G locus. The child's mother also had a normal phenotype, being homozygous for the mutation at the p.V470M locus and heterozygous for the mutation at the p.E217G locus. According to the Variant Classification Guidelines of the American College of Medical Genetics and Genomics (ACMG), p.E217G has been classified as a variant of uncertain significance, and p.V470M has been rated as a benign variant. The result of the child's sweat chloride test was negative.

Although the result of the child's sweat chloride test was negative, considering the child's acute onset, rapid disease progression, recurrent infections, and the presence of compound missense mutations in the *CFTR* gene, there is a high suspicion that the child has CF. To deeply explore the association between this case and CF, this study employed multiple mutation prediction tools such as Mutation Taster and PolyPhen-2, as well as molecular dynamics (MD) simulation techniques, to analyze the impact of the above-mentioned compound missense mutations on the function of the CFTR protein, aiming to provide new insights for the diagnosis, treatment, and genetic counseling of CF caused by the complex and diverse mutations of *CFTR*.

## 2 Materials and methods

### 2.1 Detection of *CFTR* gene mutations

The detection of CFTR gene mutations has three steps. Mutation Screening: High-throughput sequencing with an IDT chip sequences CFTR gene exons to find potential mutations. Gene Data Analysis: A multi-field-integrated genetic disease diagnosis cloud platform classifies gene variations using databases, algorithms, and the three-element and ACMG systems to identify pathogenic mutations. Verification of Suspected Pathogenic Mutations: PCR on suspected CFTR gene mutation targets, followed by Sanger sequencing on an ABI3730 sequencer and software-based confirmation of suspected mutations.

### 2.2 Prediction of mutation sites by gene mutation prediction tools

Five online tools: Mutation Taster (http://www.mutationtaster.org/) (4), PolyPhen-2 (http://genetics.bwh.harvard.edu/pph2/) (5), SIFT (https://sift.bii.a-star.edu.sg/) (6), FATHMM (https://fathmm.biocompute.org.uk/) (7), PROVEAN (http://provean.jcvi.org/index.php) (8) were employed to predict the impacts of gene mutations.

### 2.3 Prediction of wild-type and mutant CFTR protein structures via alphafold

The wild-type CFTR sequences of homo sapiens were retrieved from the Protein database on NCBI (https://www.ncbi.nlm.nih.gov/). The 3D protein structures of both the wild-type and mutant CFTR were predicted by AlphaFold 3 (https://alphafoldserver.com/) (9).

### 2.4 MD simulation and structural stability analysis

The MD simulations of CFTR were carried out following the identical simulation methods and conditions described in references (10, 11). We calculated protein root-mean-square deviation (RMSD) with “gmx rms” and radius of gyration (*R_g_*) with “gmx gyrate”, and used these as core variables to plot the free energy landscapes (FEL) in Origin 2021. For the covariance matrix calculation, we standardized simulation trajectory data with the “Bio3D” package (12) in R 4.2.2, extracted atomic coordinates by amino acid residue order, and used the built-in algorithm for matrix operations to measure residue synergistic movements.

## 3 Results

### 3.1 Case report

**Figure 1:**
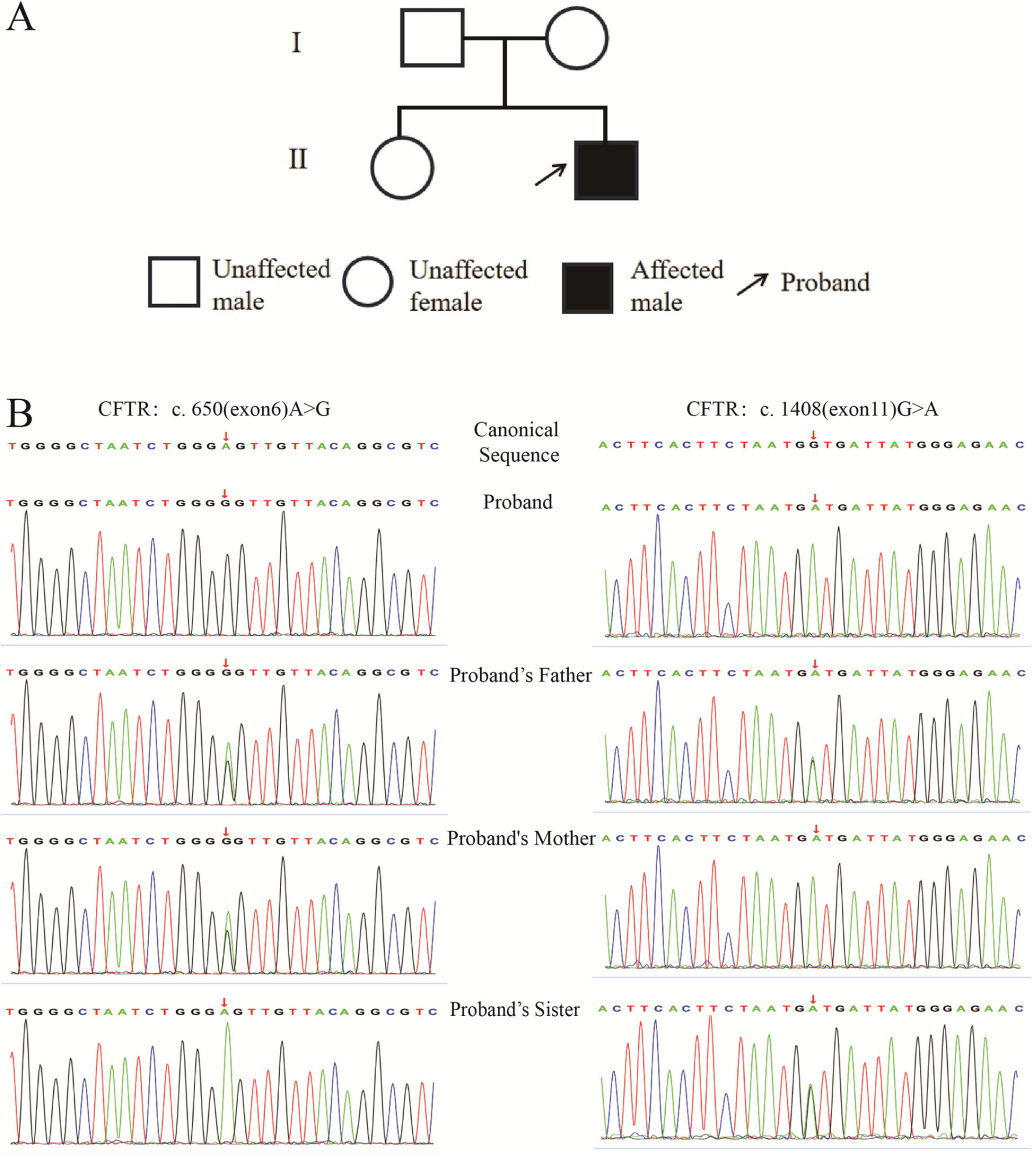
Pedigree of the proband and sequencing profiles of specific CFTR gene loci in the proband and family members. **(A)**: Pedigree of the proband; **(B)**: Sequencing profiles of CFTR gene c.650 (exon6) A>G and c.1408 (exon11) G>A sites.

The patient is a 7-month-and-24-day-old male, who was admitted to the hospital on April 23, 2023. His chief complaints were “cough and wheezing for 3 days, aggravated with dyspnea for half a day”. At 4 months old, he had suffered from acute laryngitis, grade I laryngeal obstruction, and acute bronchiolitis, and was hospitalized for 11 days before being cured and discharged. He was born full-term by spontaneous vaginal delivery, breastfed, and has a 5-year-old healthy sister. The mother has a history of sinusitis and is prone to cough, while the father has a history of allergic rhinitis. Currently, he is diagnosed with severe pneumonia, plastic bronchitis, and acute asthmatic bronchitis. There are suspicions of primary ciliary dyskinesia and CF. The pedigree chart of the proband is shown in Figure 1A.

### 3.2 Laboratory diagnosis

On admission, physical examination showed: T 36.8 °C, P 168 beats/min, R 48 breaths/min, SpO2 90%, Wt: 11 kg. The patient was conscious but irritable, with rapid breathing, nasal flaring, three-concave sign, pharyngeal congestion, rough breath sounds in both lungs, scattered wheezing sounds, no breath sound in the right upper lung, strong heart sounds, regular heart rate, warm extremities, and no abnormalities in abdominal and nervous system examinations. Laboratory test results are presented in Table 1. In addition, liver and kidney functions, myocardial enzymes, and electrolytes were normal. The total protein was 54.9 g/L (reference range: 55–75 g/L), albumin was 36.6 g/L (reference range: 39–52 g/L), and triglyceride was 0.42 mmol/L (reference range: 0.6–1.71 mmol/L). In the five-item test of blood coagulation function, D-dimer was 0.72 mg/L (reference range: 0–0.5 mg/L), and the rest were normal. The patient had type A Rh-positive blood. The T-lymphocyte test for tuberculosis infection was negative. Hepatitis markers, HIV, syphilis, EB virus antibody quantification were all negative. The galactomannan test for Aspergillus (GM test) was 0.187 ug/L (negative), and the fungal (1,3) β-D-glucan test (G test) was <37 pg/ml (negative). The antigens of influenza A and B viruses were both negative. Humoral immunity and complement were normal. Sputum tests: Deep sputum culture was positive for Pseudomonas aeruginosa twice, while sputum fungal and tuberculosis culture were negative. Imaging and other tests: Chest CT on admission showed a patchy dense consolidation shadow in the right upper lobe of the lung, without air-bronchogram sign. The right horizontal fissure was slightly elevated. Airway reconstruction suggested occlusion of the right upper lobe bronchus and slightly narrow lower lobe bronchus, indicating consolidation of the right upper lobe of the lung and occlusion of the right upper lobe bronchus. Cardiac ultrasound and abdominal ultrasound (including liver, gallbladder, spleen, pancreas, urinary system, and gastrointestinal tract) were normal. After admission, due to respiratory failure, the patient underwent emergency bronchoscopy and treatment under endotracheal intubation. BALF tNGS showed Bocavirus type I 64,029 × 10^6^; BALF pathogen DNA tests for multiple bacteria were negative. The patient received a series of treatments including ventilator-assisted ventilation, anti-infection, anti-inflammation, etc. The condition gradually improved, and the patient was discharged on the 17th day after admission. After discharge, there were follow-up re-examinations and treatments. Considering the acute onset, rapid progression, and gene test results, a tendency of CF was suspected.

**Table 1:** Summary of laboratory test results for the pediatric patient.

Test Items	Results	Reference values	Units
Arterial blood gas analysis			
pH	7.3	7.35–7.45	/
pCO₂	44.3	35.0–45.0	mmHg
pO₂	117	83.0–108	mmHg
Complete blood count			
WBC	9.79 × 10⁹	/	/L
Neutrophil percentage	52.30%	/	%
Lymphocyte percentage	33.30%	/	%
Eosinophil percentage	6.80%	/	%
Hb	120	/	g/L
Plt	396 × 10⁹	/	/L
CRP	6	0–10	mg/L
PCT	<0.02	0–0.1	ng/ml
Cytokines			
IL-2	5.61	0–9.8	pg/ml
IL-4	2.08	0–3	pg/ml
IL-6	8.3	0–16.6	pg/ml
IL-10	14.37	0–4.9	pg/ml
IL-17A	0.82	0–20.6	pg/ml
TNF	0.84	0–5.2	pg/ml
γ-IFN	7.51	0–17.3	pg/ml
Lymphocyte subsets			
CD3	30.95%	55.32–73.11%	%
CD4	18.76%	2.178–47.74%	%
CD8	12.46%	15.88–31.48%	%
CD56	8.58%	5.67–15.9%	%
CD19	58.96%	17.2–29.71%	%
CD4/CD8 ratio	1.51	0.93–2.52	/
Lymphocyte count	616	3730–8760	/μl
Total T lymphocyte count	191	2187–6352	/μl
CD4+ T lymphocyte count	191	1125–3768	/μl
CD8+ T lymphocyte count	77	686–2278	/μl
NK cell count	52	306–896	/μl
B lymphocyte count	361	916–1832	/μl

### 3.3 Analysis of *CFTR* gene mutations

#### 3.3.1 Results of trio whole-exome sequencing for the pedigree

Trio whole-exome sequencing for this pedigree are shown in Figure 1B. The figure presents the sequencing peak diagrams of two loci of the CFTR gene in the proband and his family members. Firstly, regarding the mutation locus of p.E217G, the proband is homozygous for this mutation, the father is heterozygous, the mother is heterozygous, and the sister is homozygous wild-type. Secondly, for the mutation locus of p.V470M, the proband is homozygous for this mutation, the father is heterozygous, the mother is homozygous, and the sister is heterozygous.

#### 3.3.2 Mutation site prediction using genetic mutation prediction tools

As shown in Table 2, Mutation Taster predicted that the CFTR E217G mutation had a pathogenic result, while the CFTR V470M mutation was predicted to be a gene polymorphism. PolyPhen-2 predicted that both the CFTR E217G mutation and the CFTR V470M mutation had a benign result. SIFT predicted that both CFTR E217G and CFTR V470M were deleterious. FATHMM also predicted that both were deleterious. In contrast, PROVEAN predicted that both CFTR E217G and CFTR V470M were neutral genes.

**Table 2:** Summary of prediction results of different prediction tools for CFTR V470M and CFTR E217G mutations.

Predict tool	CFTR V470M	CFTR E217G
Mutation taster	Polymorphism	Disease causing
polyPhen-2	Benign	Benign
SIFT	Deleterious	Deleterious
FATHMM	Damaging	Damaging
PROVEAN	Neutral	Neutral

### 3.4 MD simulation

**Figure 2:**
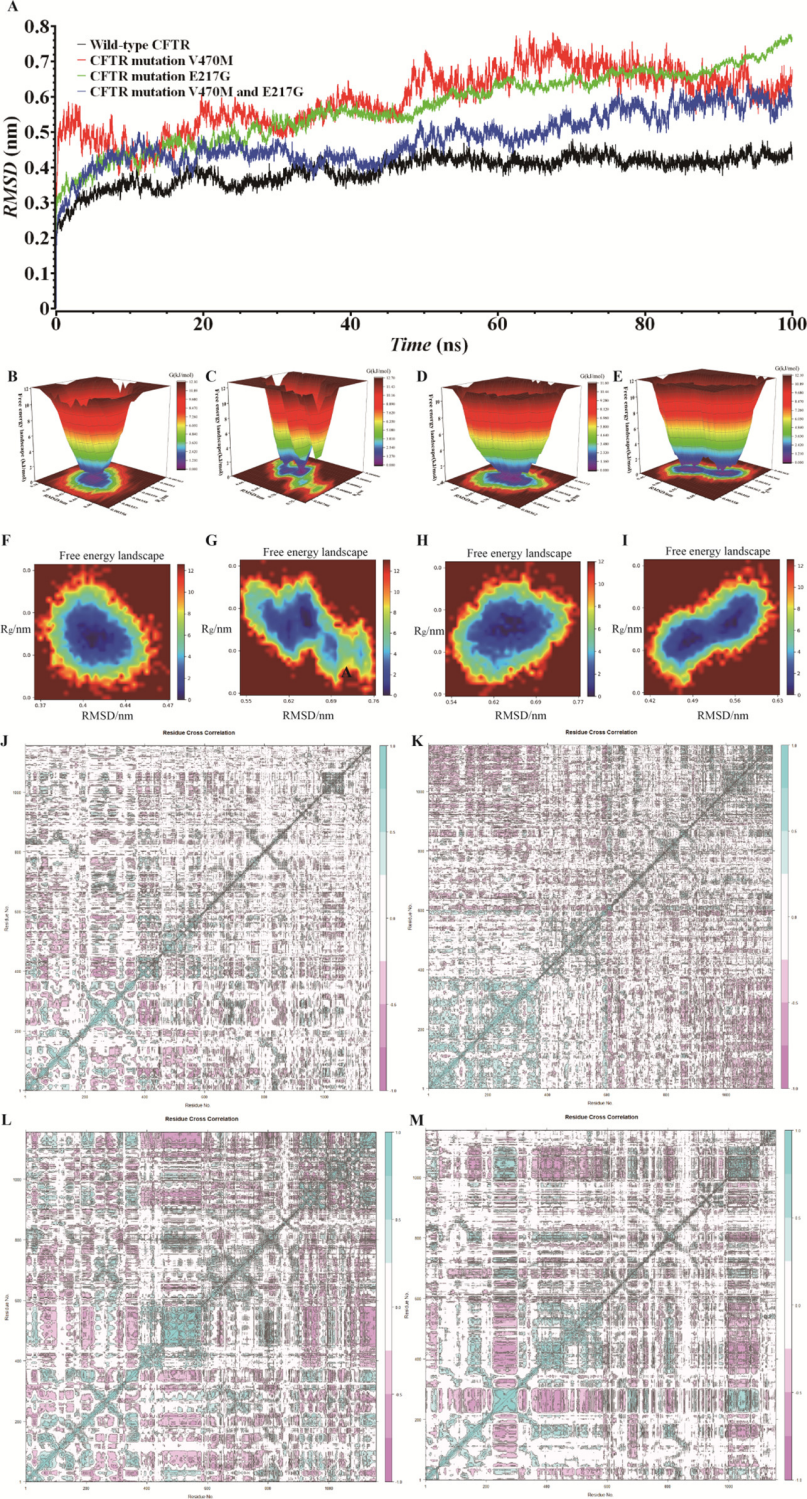
Md simulation and covariance matrix analysis: unveiling the structural stability of wild-type and mutant CFTR proteins. **(A)** RMSD analysis of wild-type CFTR, CFTR V470M, CFTR E217G, and CFTR V470M/E217G double-mutant based on MD simulations. **(B–I)** FEL of CFTR proteins under different conditions. **(B,F)** Wild-type CFTR; **(C,G)** CFTR E217G mutant; **(D,H)**: CFTR V470M mutant; **(E,I)** CFTR V470M/E217G double-mutant. **(J)** Wild-type CFTR protein; **(K)** CFTR E217G mutant; **(L)** CFTR V470M mutant; **(M)** CFTR V470M and E217G double-mutant.

We employed MD simulation methods to analyze the impact of CFTR mutations on protein structure and function. RMSD results (Figure 2A) revealed that the RMSD value of the wild-type CFTR protein gradually converged and stabilized during the simulation, indicating a relatively stable structure. Although the RMSD curve of the CFTR V470M single-mutant exhibited fluctuations, it showed an overall convergence trend. However, the RMSD curves of the CFTR V470M and E217G double-mutant similar to the proband and the CFTR E217G single-mutant protein did not show obvious convergence and equilibrium, and the protein structures were continuously in a state of significant fluctuations. This indicates that CFTR gene mutations similar to those of the proband significantly reduce protein stability. An unstable protein structure may impede its correct folding and maintenance of a normal conformation, thereby having a negative impact on the normal function of the CFTR protein.

Figures 2B–I present the FELs of the CFTR protein under different conditions. The wild-type (Figures 2B,F) exhibits a single, deep energy well, indicating high structural stability during simulation, with the low-free-energy conformation being dominant and the structure being relatively stable. For the E217G mutant (Figures 2C,G), the distribution and depth of the energy wells are altered, and multiple relatively stable sub-states exist. The V470M mutant (Figures 2D,H) has a special energy distribution. The V470M and E217G double-mutant (Figures 2E,I) has the most complex energy well. Compared with the wild-type and single mutants, significant differences are observed in the number, position, and depth of its energy wells, suggesting the presence of multiple relatively unstable conformational states. This is highly likely to have a negative impact on the structure, stability, and normal function of the CFTR protein.

We plotted the covariance matrix to investigate the structural stability of the CFTR protein. As shown in Figure 2J, the covariance matrix of the wild-type CFTR protein exhibits a specific element distribution, indicating that the amino acid residues have stable synergistic movement during structural changes. Figure 2K reveals that the matrix element distribution of the E217G mutant is different from that of the wild type, suggesting that the mutation alters the synergistic movement of residues, affecting the internal interactions and stability of the protein. Figure 2L demonstrates that the matrix element distribution of the V470M mutant differs from both that of the wild type and the E217G mutant, indicating a unique impact on the synergistic movement of residues and a distinct mechanism of action. Figure 2M indicates that the matrix element distribution of the V470M and E217G double mutant is the most complex, severely interfering with the synergistic movement of residues, making the protein structure more unstable and potentially leading to the loss of its normal function.

### 3.5 Treatment and follow-up

Upon admission, the patient was diagnosed with acute type I respiratory failure, severe pneumonia, atelectasis, and plastic bronchitis. Immediate medical interventions were carried out, including endotracheal intubation, ventilator-assisted ventilation, intravenous infusion of cefazolin combined with erythromycin for anti-infection, intravenous administration of methylprednisolone for anti-inflammation, and symptomatic treatments like atomization and sputum suction. On the second day, the child had no breath sounds in the right upper lung, was in poor condition, and the pneumonia didn't absorb well. Another bronchoscopy showed acute and chronic inflammation in both lungs, especially in some lobes, along with plastic bronchitis. On the third day, antibiotics were adjusted, and immunoglobulin was infused. On the fourth day, a third bronchoscopy was done, showing more severe inflammation in the right lung. Thanks to the treatments, the patient's condition improved. On the sixth day, the ventilation mode changed, and the dosage of methylprednisolone was reduced. On the eighth day, the patient switched to nasal cannula oxygen inhalation. On the twelfth day, sputum culture showed Pseudomonas aeruginosa, and the anti-infection drug was changed. On the seventeenth day, the patient was discharged. After discharge, anti-infection and atomization treatments were continued. In June 2023, re-examination showed lung lesions, bronchial stenosis, and pulmonary function dysfunction. Four months after discharge, the sweat chloride test was negative. Considering infantile asthma, atomization treatment was given for seven months. In 2024, the child had wheezing, gastroenteritis, and mycoplasma pneumonia, all cured through outpatient treatment. Although the result of the child's sweat chloride test was negative, considering the child's acute onset, rapid disease progression, recurrent infections, and the presence of compound missense mutations in the CFTR gene, there is a high suspicion that the child has CF.

## 4 Discussion

In the current research field of CF, the understanding and research of this disease are constantly making new progress. From the perspective of global epidemiological data, approximately 162,428 people are affected by CF. However, it is concerning that 35% of these patients remain undiagnosed. This phenomenon is observed in 94 countries, fully reflecting the extensive global distribution of CF and the significant challenges in its diagnosis. Currently, the diagnosis of CF caused by CFTR gene mutations primarily relies on genetic testing and the sweat chloride test.

The p.E217G has been classified as a variant of uncertain significance according to the ACMG Variant Classification Guidelines. This variant has been included in the dbSNP database (rs121909046) (13). Its frequencies in databases of carrier frequencies among the general population, such as the 1000 Genomes Project and gnomAD, are relatively high, reaching 0.0144 and 0.0045 respectively (14). Moreover, in the gnomAD database, 29 normal individuals are homozygous carriers of this variant, with the majority (24 out of 29) being from the Finnish subpopulation in Europe. There are also 2 normal individuals with homozygous carriage in the East Asian population. Additionally, this variant has been detected in patients with multiple CFTR-related diseases, including cystic CF, chronic pancreatitis, and congenital bilateral absence of the vas deferens (CBAVD) (15–17). The p.V470M variant, on the other hand, has been rated as a benign variant by the ACMG Variant Classification Guidelines. Regarding the p.E217G variant, it is located in the loop region of the transmembrane domain of CFTR. Krainer et al. explored its structural effects using single-molecule FRET analysis and found that this mutation generates an additional GXXXG helix-helix interaction motif in the hairpin, leading to a conformational change in the transmembrane helical segments of CFTR. Consequently, this results in misfolding and functional defects of CFTR. However, the small molecule corrector Lumacaftor can not only stabilize the E217G mutant hairpin but also has a helix-stabilizing effect on the transmembrane helical hairpins of the wild-type CFTR and other mutant hairpins (18). Hämmerle et al. confirmed that the E217G mutation disrupts the stability of the CFTR ion channel (19). Lee et al. demonstrated in a case-control study that the E217G variant is associated with bronchiectasis and chronic pancreatitis. Moreover, the common polymorphism of M470V affects the strength of the disease association. For example, in the two haplotypes with IVS8 T5, the T5-V470 haplotype has a stronger disease association than the T5-M470 haplotype. The M470-E217G mutation reduces the membrane protein expression and chloride ion transport activity by 60%–70% (20). In 2020, Petrova et al. detected a compound heterozygous variant of p.E217G and p.F508del in a Russian patient with CF. The patient's sweat chloride level was 63 mmol/L. This indicates that the complex allele E217G-M470;F508del may be the cause of the patient's clinical phenotype. In-depth research on these variants will contribute to a further understanding of the pathogenesis of CF, providing important evidence for the diagnosis, treatment, and prevention of the disease.

## 5 Conclusion

Given the complex condition of the patient, although the result of the child's sweat chloride test was negative, considering the child's acute onset, rapid disease progression, recurrent infections, and the presence of compound missense mutations in the CFTR gene, we have a high suspicion that the child has CF. In this study, we attempted to use computational chemistry methods such as mutation prediction tools and MD simulations to explore how mutations reshape the 3D structure of proteins, thereby leading to functional changes. This exploration provides a microscopic and dynamic perspective for the study of the mutation mechanisms of CFTR. However, we are well aware that these predictions and simulations still have a long way to go compared to real-world research and can only offer some preliminary thoughts and inspirations for disease diagnosis, formulation of treatment plans, and genetic counseling. Looking ahead, we plan to carry out work related to the synthesis and purification of the mutated CFTR protein, with the expectation of further analyzing the effects of mutations on aspects such as protein structure, stability, folding processes, interactions with other molecules, and ion channel functions. We understand that this is just a small step on the path of exploring the association between CFTR mutations and the occurrence and development of the disease, and we hope that it can provide some references for the subsequent development of treatment strategies and the improvement of the genetic counseling system.

## Data Availability

The original contributions presented in the study are included in the article/Supplementary Material, further inquiries can be directed to the corresponding author.
